# Supplementary figures and images for: The specific features of the developing T cell compartment of the neonatal lung are a determinant of respiratory syncytial virus immunopathogenesis
Source: PLoS Pathog. 2021 Apr 28;17(4):e1009529. doi: 10.1371/journal.ppat.1009529 (PMC8109812; doi:10.1371/journal.ppat.1009529)

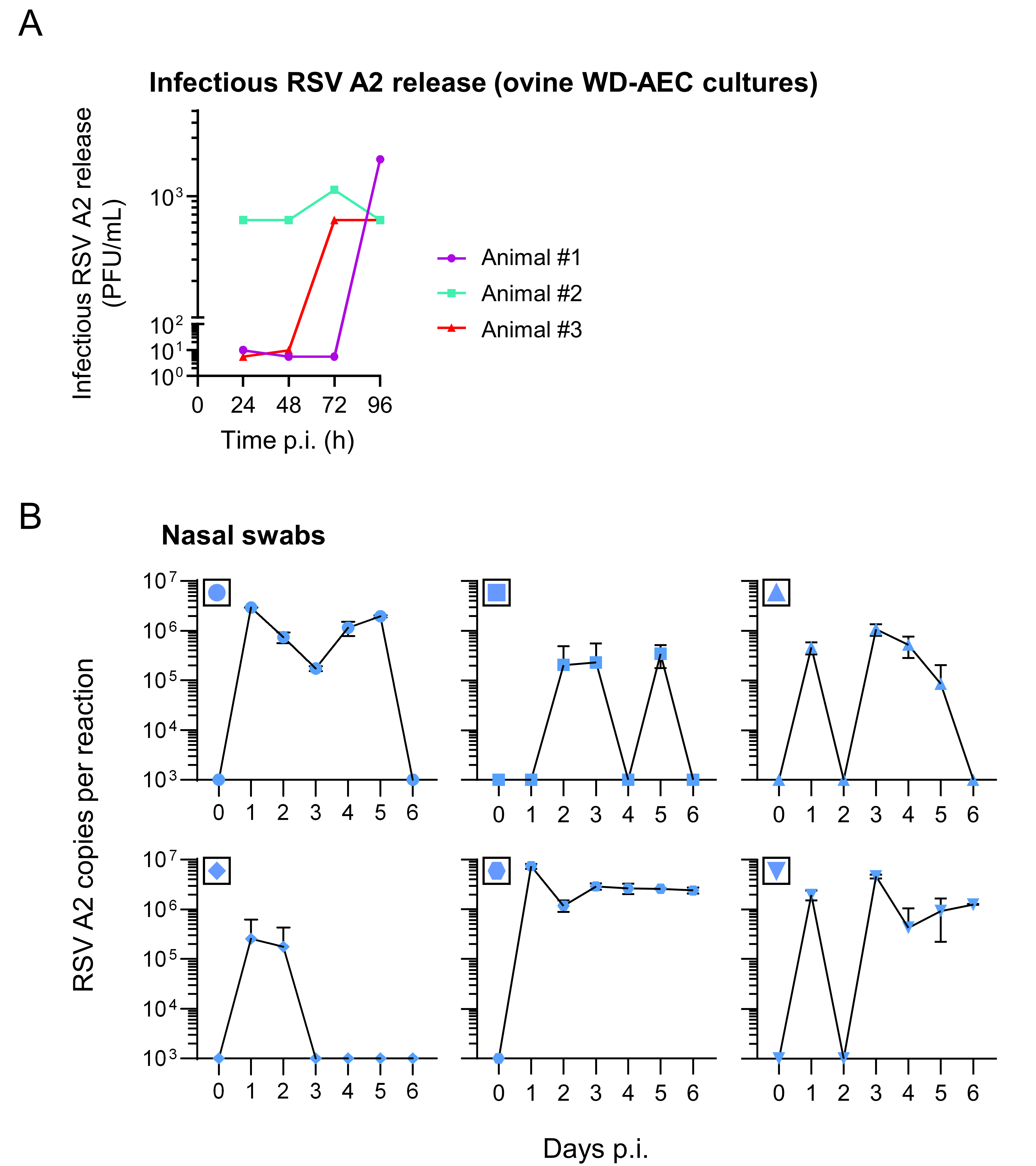

Supplement: S1 Fig — (A) Ovine WD-AEC cultures from 3 independent animals were infected with RSV A2 (MOI = 1 PFU/cell). Apical washes were harvested at different time points (24, 48, 72 and 96 h p.i.) and titrated on HEp-2 cells. (B) Nasal swabs from RSV A2-infected neonates were taken daily on a 6 day-duration period. RSV A2 was then quantified by qPCR. Each sample was measured in duplicate. After the initial decrease of RSV A2 titers, a second peak was detected in 5 out of 6 animals, showing the capacity of RSV A2 to replicate in vivo in the ovine model. (TIF) [file ppat.1009529.s001.tif]

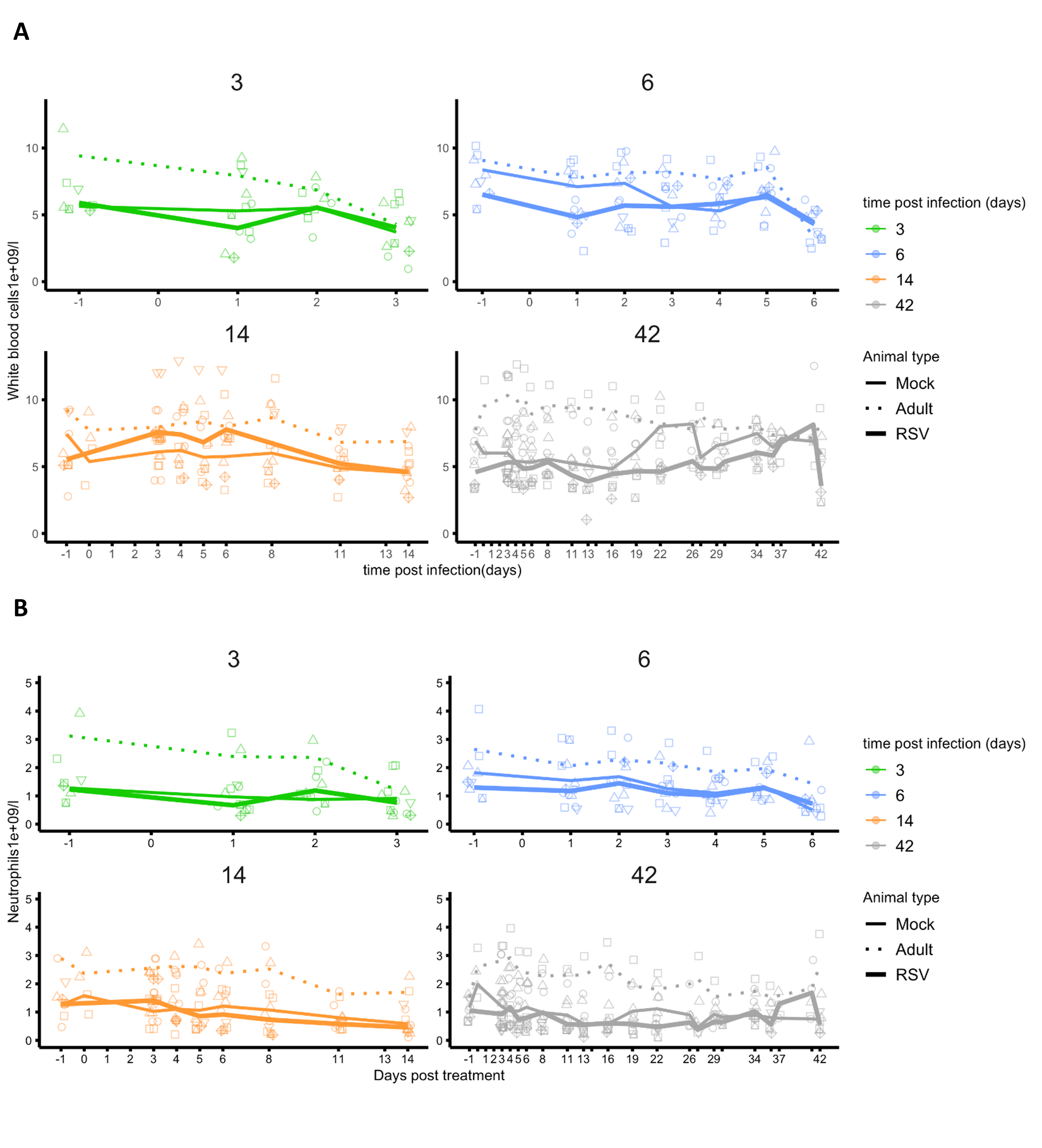

Supplement: S2 Fig — Frequencies in the peripheral blood of (A) White blood cells (WBC) and (B) polymorphonuclear leukocytes (PMNs) over the course of RSV A2 disease. Data were obtained with VETSCAN HM5 Hematology Analyzer (Abaxis) and graphs were generated with R v.3.4.4 (2018-03-15). (TIF) [file ppat.1009529.s002.tif]

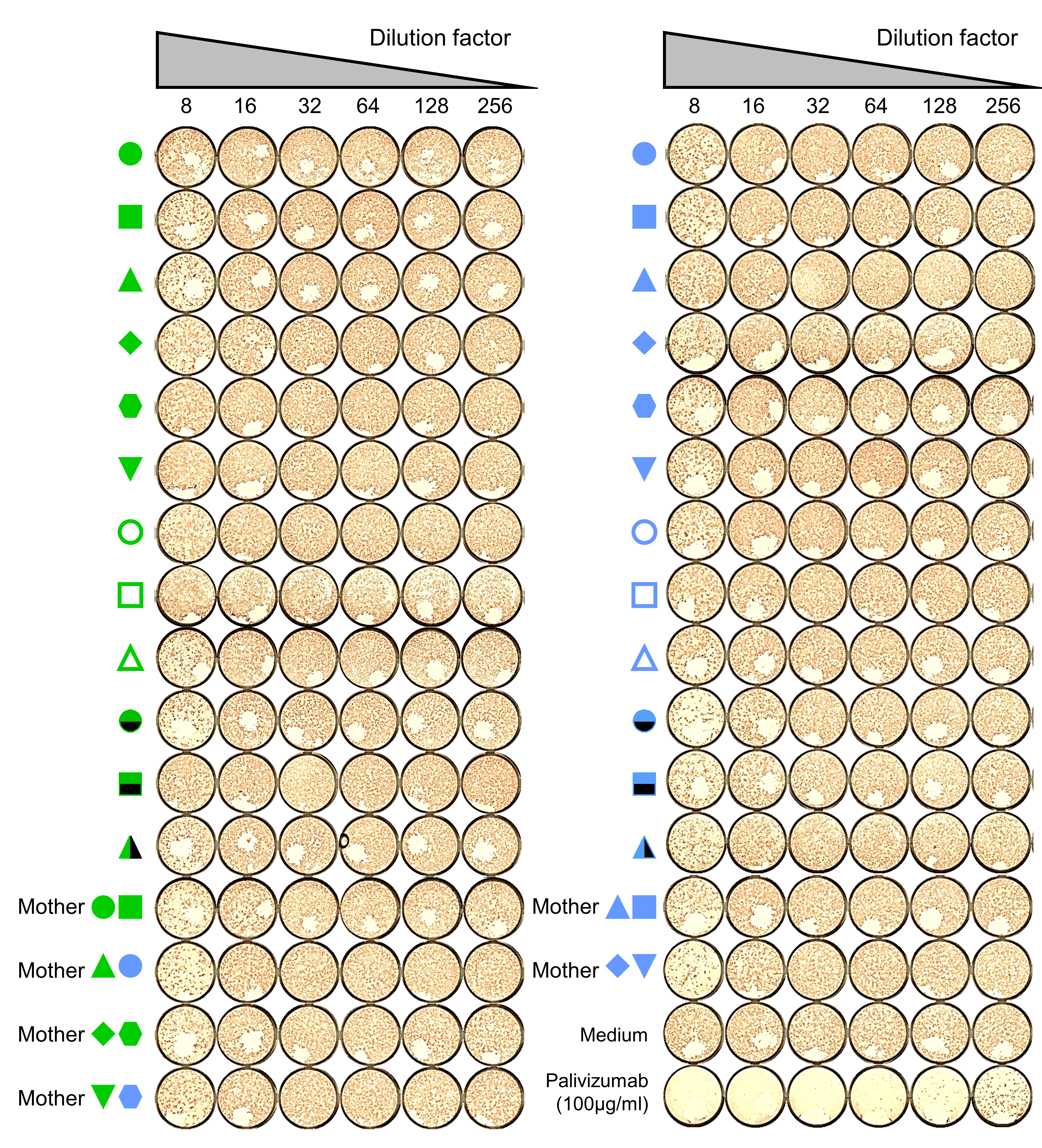

Supplement: S3 Fig — Serums collected in animals prior RSV A2 infection (baseline) were co-incubated with RSV A2 (100 PFU) and applied to HEp-2 cells for 48 hours. The results show that all tested animals were negative. Cell culture medium and Palivizumab were used as negative and positive controls, respectively. (TIF) [file ppat.1009529.s003.tif]

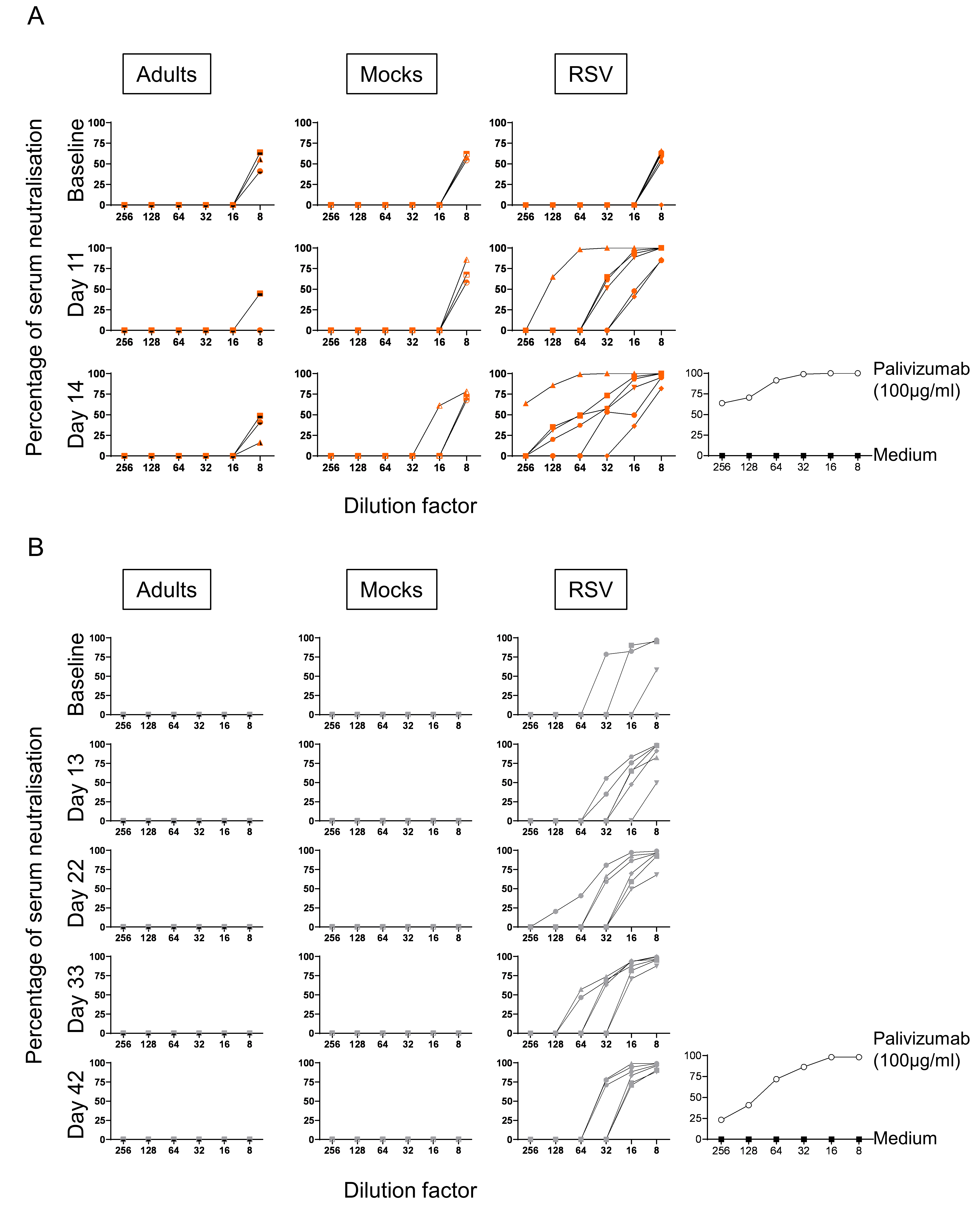

Supplement: S4 Fig — Serums collected in animals prior RSV A2 infection (baseline) or at different time points as indicated, were co-incubated with RSV A2 (100 PFU) and applied to HEp-2 cells for 48 hours. RSV-infected neonates have naturally acquired NAb at day 13–14 p.i. (A), that persist over a 42-day long period (B). Each symbol represents an individual animal (symbols filled with black, healthy adults; transparent symbols, mock neonates; solid color symbols, neonates infected with RSV). Cell culture medium and Palivizumab were used as negative and positive controls, respectively. (TIF) [file ppat.1009529.s004.tif]

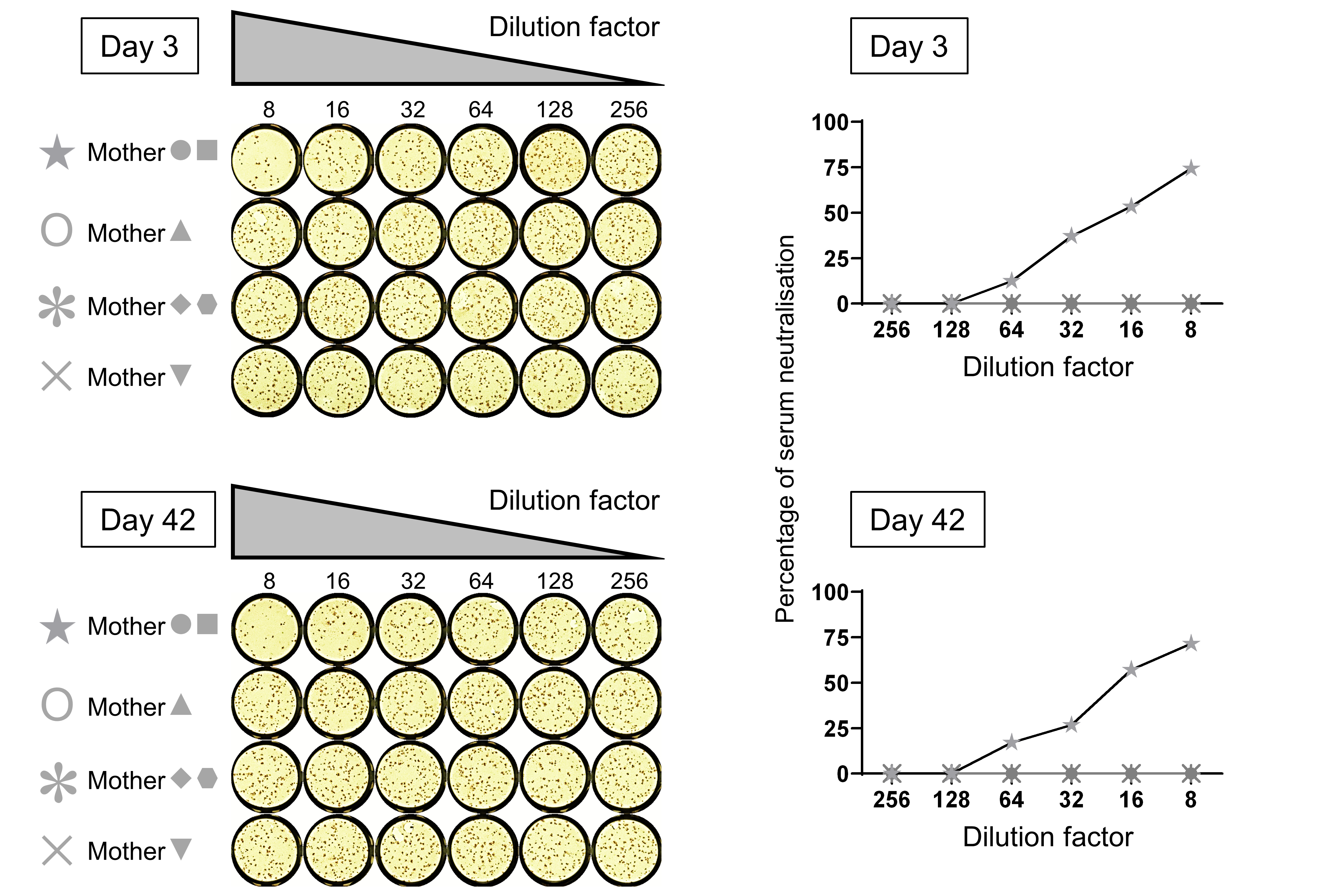

Supplement: S5 Fig — Serums collected in animals at the initial phase of RSV A2 infection (day 3) or at day 42 p.i., were co-incubated with RSV A2 (100 PFU) and applied to HEp-2 cells for 48 hours. The mothers of two RSV-infected sibling presented an RSV-neutralization over the 42-day long period of the experiment. Each symbol represents an individual animal. (TIF) [file ppat.1009529.s005.tif]

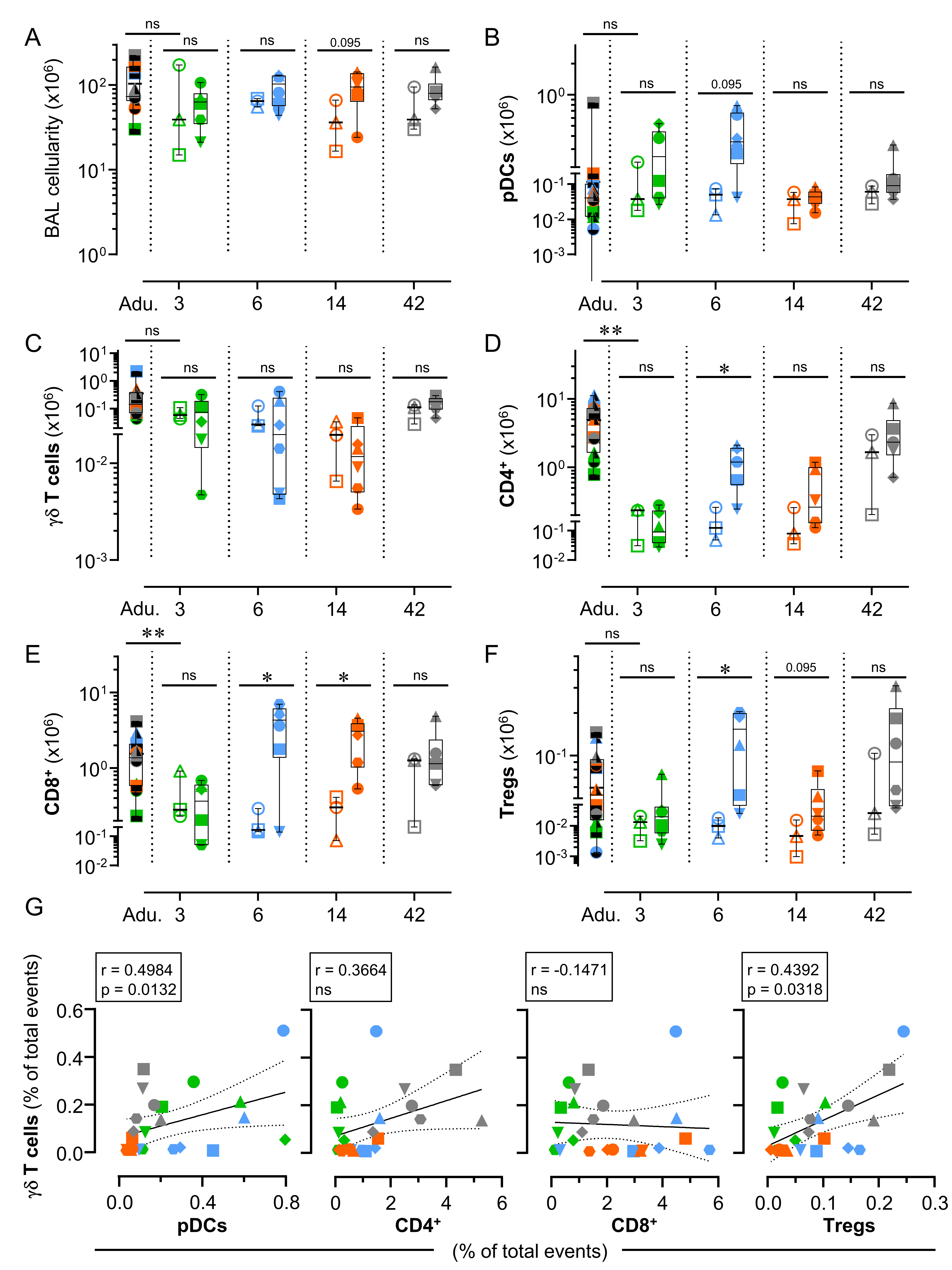

Supplement: S6 Fig — (A) Total cellularity measured in the BALs. Cell count and viability was done with Trypan Blue exclusion dye. (B-F) Absolute cell counts of pDCs (B), γδ T cells (C), CD4+ T cells, (D), CD8+ T cells (E) and Tregs (F). Each symbol represents an individual animal (healthy adults, n = 12; mock neonates, n = 3 per time point; neonates infected with RSV, n = 6 per time point). Boxplots indicate median value (center line) and interquartile ranges (box edges), with whiskers extending to the lowest and the highest values. Groups were compared using Mann–Whitney U-tests (A-F). Stars indicate significance levels. *, p < 0.05; **, p < 0.01; ***, p < 0.001. (G) Correlation coefficient (r) obtained in BALs with γδ T cells (% of total events) calculated as a function of pDCs (% of total events), CD4+ T cells, (% of total events), CD8+ T cells (% of total events) and Tregs (% of total events). Absence of negative correlation shows that the γδ T-cell depletion observed in Fig 3A is not related to the expansion of another immune cell subset. (TIF) [file ppat.1009529.s006.tif]

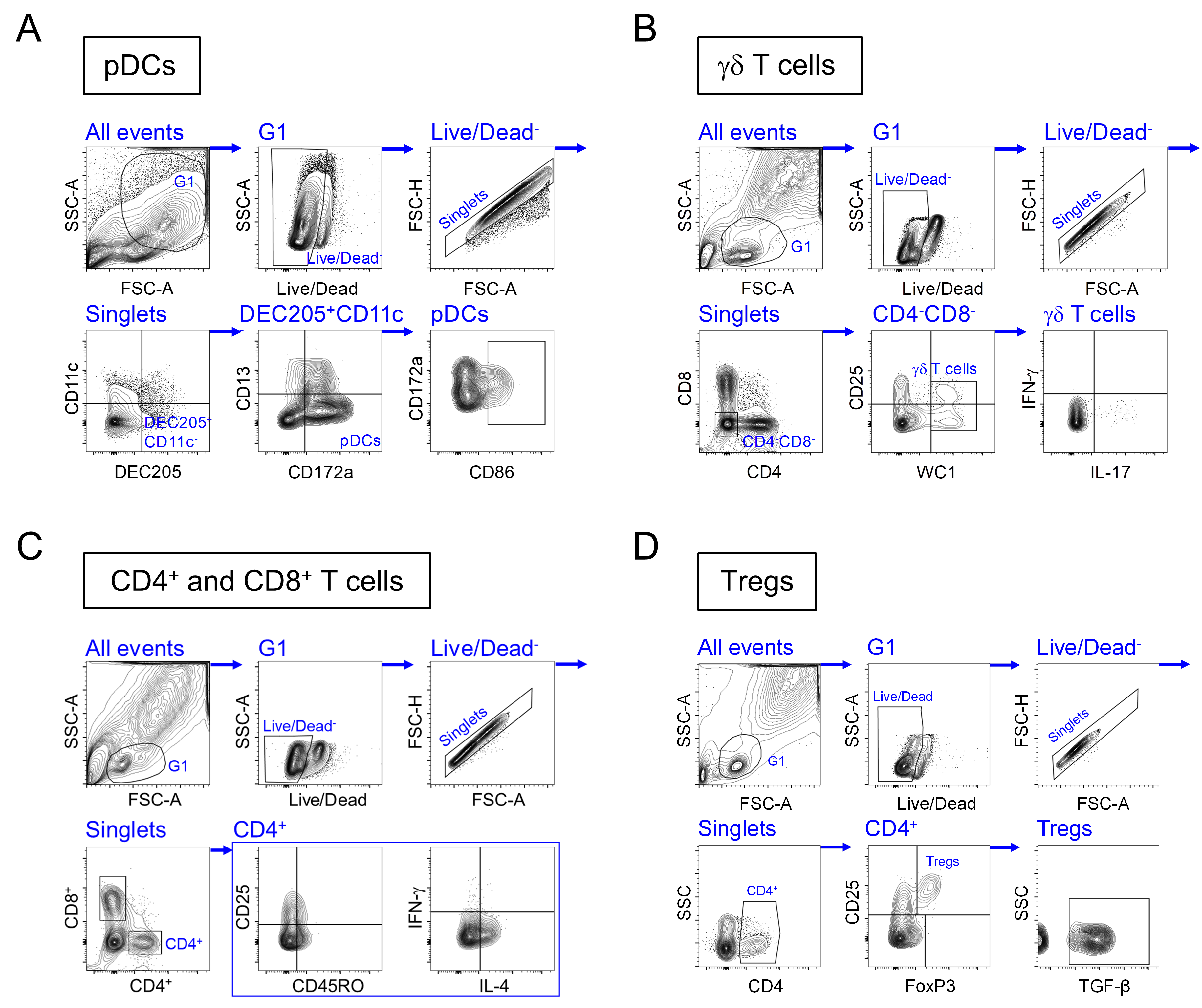

Supplement: S7 Fig — Example of gating strategy for multiparameter FCM analysis of ovine pDCs (A), γδ T cells (B), CD4+ and CD8+ T cells (C) and Tregs (D). (TIF) [file ppat.1009529.s007.tif]

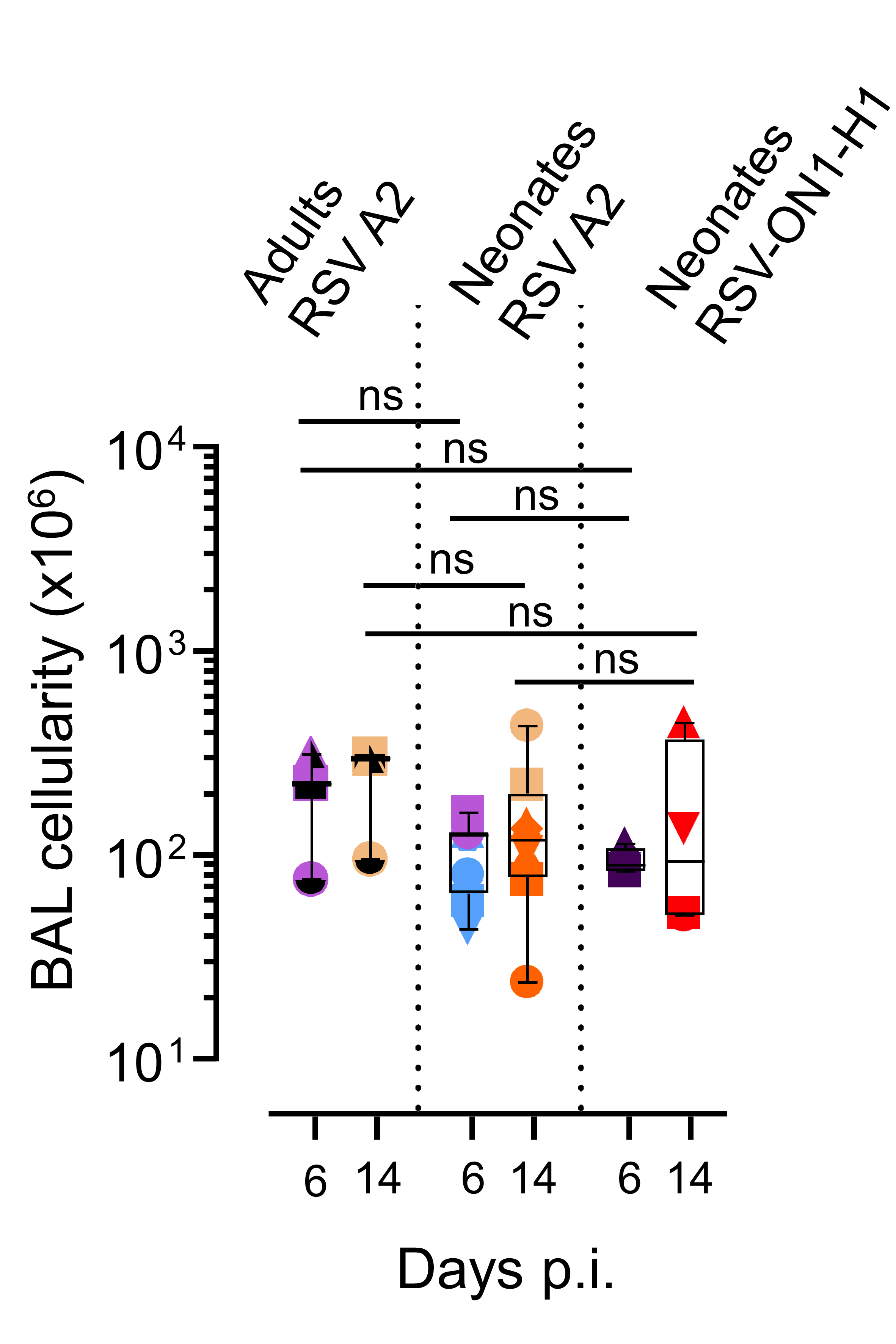

Supplement: S8 Fig — Cell count and viability was done with Trypan Blue exclusion dye. Each symbol represents an individual animal (RSV A2 infected adults, n = 3 per time point; neonates infected with RSV A2, n = 8 per time point; neonates infected with RSV-ON1-H1, n = 4 per time point). Boxplots indicate median value (center line) and interquartile ranges (box edges), with whiskers extending to the lowest and the highest values. Groups were compared using one-way ANOVA followed by Turkey’s post hoc test. (TIF) [file ppat.1009529.s008.tif]

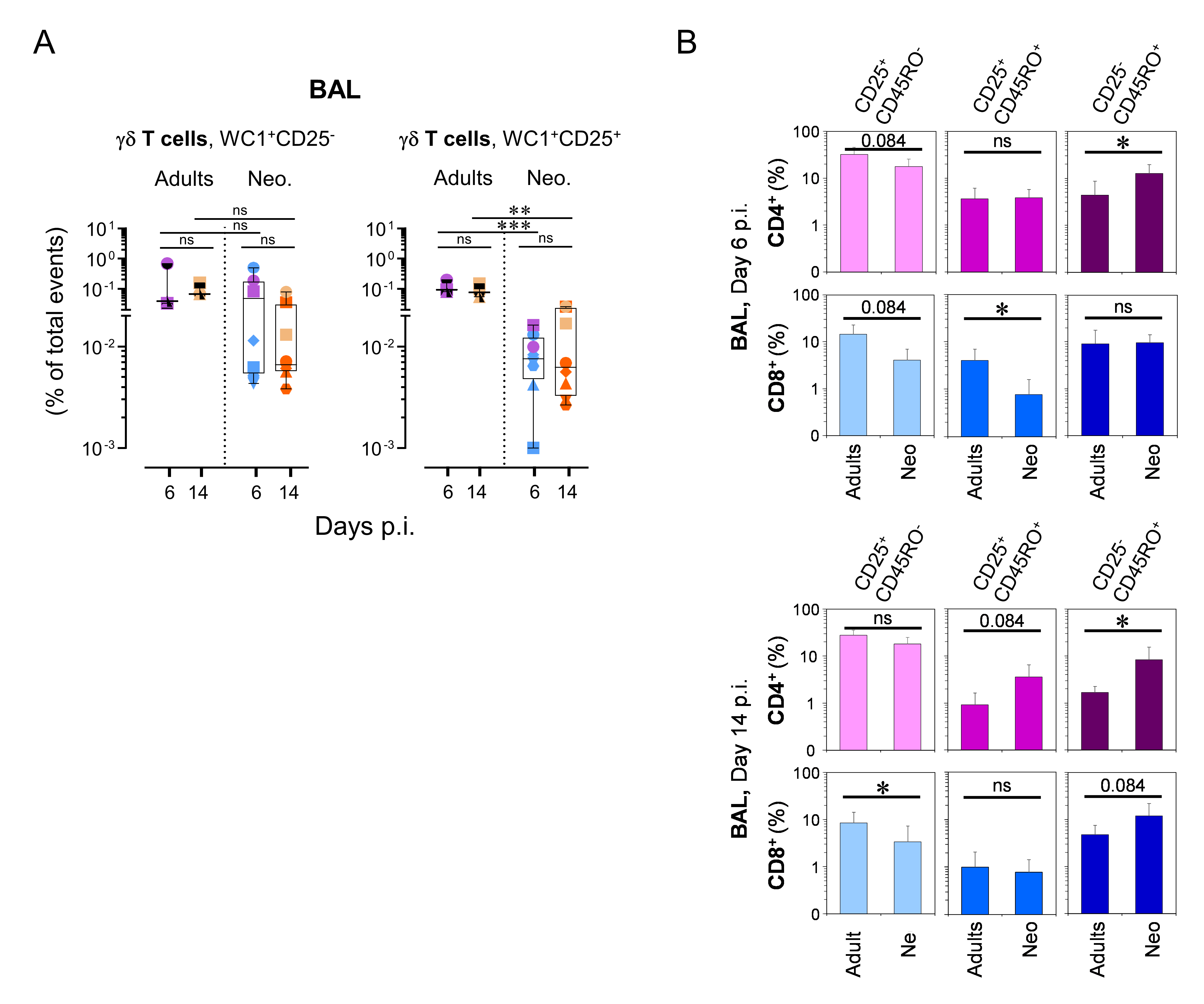

Supplement: S9 Fig — (A) As in Fig 7G, but with plots integrating all animals. Each symbol represents an individual animal (RSV A2 infected adults, n = 3; Neonates infected with RSV A2, n = 8 per time point). Boxplots indicate median value (center line) and interquartile ranges (box edges), with whiskers extending to the lowest and the highest values. Groups were compared using one-way ANOVA followed by Turkey’s post hoc test. (B) As in Fig 7K and 7O, but with histograms integrating all individuals. Groups were compared using Mann–Whitney U-tests. Stars indicate significance levels. *, p < 0.05. (TIF) [file ppat.1009529.s009.tif]
