# Supplementary material for: The specific features of the developing T cell compartment of the neonatal lung are a determinant of respiratory syncytial virus immunopathogenesis
Source: PLoS Pathog. 2021 Apr 28;17(4):e1009529. doi: 10.1371/journal.ppat.1009529 (PMC8109812; doi:10.1371/journal.ppat.1009529)
Supplement: S1 Table — (DOCX) [file ppat.1009529.s010.docx]

| **Antibody** | **Dilution** | **Host, isotype** | **Clone** | **Reference, Source** |
| --- | --- | --- | --- | --- |
| Anti-DEC205 | 1:20 | Mouse, IgG2b | CC98 | MCA1651GA, BioRad |
| Anti-CD11c | 1:33 | Mouse, IgM | BAQ153A | WS0519B-100, Kingfisher |
| Anti-CD172a | 1:400 | Mouse, IgG1 | DH59B | WS0567B-100, Kingfisher |
| Anti-CD13 | 1:100 | Mouse, IgG1 | CC81 | MCA2338GA, BioRad |
| Anti-CD86-PE | 1:10 | Mouse, IgG1 | IL-A190 | MCA2437PE, Bio-Rad |
| Anti-IL-12/23 | 1:80 | Mouse, IgG2a | CC301 | MCA1782EL, Bio-Rad |
| Anti-CD45RO-PE | 1:10 | Mouse, IgG3 | IL-A116A | MCA2434PE, Bio-Rad |
| Anti-CD4 | 1:400 | Mouse, IgG2a | 44.38 | MCA2213GA, Bio-Rad |
| Anti-CD8 | 1:160 | Mouse, IgG1 | CC58 | MCA1654G, Bio-Rad |
| Anti-CD25 | 1:200 | Mouse, IgG3 | LCTB2A | WS0597B-100, Kingfisher |
| Anti-IFN-γ-AF647 | 1:160 | Mouse, IgG1 | CC302 | MCA1783A647, Bio-Rad |
| Anti-IL-4-FITC | 1:10 | Mouse, IgG2a | CC303 | MCA1820F, Bio-Rad |
| Anti-FOXP3-FITC | 1:50 | Rat, IgG2a | FJK-16s | 11-5773-82, TermoFisher |
| Anti-TGF-β | 1:100 | Mouse, IgG1 | TB21 | MCA797, Bio-Rad |
| Anti-IL-10 | 1:40 | Mouse, IgG2b | CC318 | MCA2110, Bio-Rad |
| Anti-WC1-FITC | 1:80 | Mouse, IgG2a | CC15 | MCA838F, Bio-Rad |
| Anti-IL-17A-PE | 1:20 | Mouse, IgG1 | Ebio64DEC17 | 12-7179-42, ThermoFisher |
| Anti-mouse IgG1, FITC | 1:1000 | Goat, IgG | - | A-21121, ThermoFisher |
| Anti-mouse IgG1, PE | 1:200 | Goat, IgG | - | P-21129, ThermoFisher |
| [Anti-mouse IgG1, PerCP-Cy5.5](https://datasheets.scbt.com/sc-45105.pdf) | 1:80 | Rat, IgG | RMG1-1 | 406612, Biolegend |
| Anti-Mouse IgG3, PerCP-Cy5.5 | 1:200 | Goat, IgG | - | 1100-13, SouthernBiotech |
| [Anti-mouse IgM, PerCP-Cy5.5](https://datasheets.scbt.com/sc-45105.pdf) | 1:20 | Rat, IgG2a | RMM-1 | 406512, Biolegend |
| Anti-mouse IgG1, PE-Cy7 | 1:640 | Rat, IgG | RMG1-1 | 406614, Biolegend |
| Anti-mouse IgG2b, PE-Cy7 | 1:250 | Goat, IgG | - | 1090-17, SouthernBiotech |
| Anti-mouse IgG1, a647 | 1:1000 | Goat, IgG | - | A-21240, ThermoFisher |
| Anti-mouse IgG2b, a647 | 1:1000 | Goat, IgG | - | A-21242, ThermoFisher |
| Anti-mouse IgG1, APC-Cy7 | 1:40 | Rat, IgG | RMG1-1 | 406620, BioLegend |
| Anti-mouse IgG2a, APC-Cy7 | 1:250 | Goat, IgG | - | 1080-19, SouthernBiotech |
| Anti-mouse IgG1, BV421 | 1:80 | Rat, IgG | RMG1-1 | 406616, BioLegend |
