# Supplementary material for: The specific features of the developing T cell compartment of the neonatal lung are a determinant of respiratory syncytial virus immunopathogenesis
Source: PLoS Pathog. 2021 Apr 28;17(4):e1009529. doi: 10.1371/journal.ppat.1009529 (PMC8109812; doi:10.1371/journal.ppat.1009529)
Supplement: S2 Table — (DOCX) [file ppat.1009529.s011.docx]

| **Subset** | **Live/Dead Aqua** | **Surface**  **1** | **Surface**  **2** | **IgG block** | **Surface**  **3** | **Fix & Perm** | **Intracell.**  **1** | **Intracell.**  **2** |
| --- | --- | --- | --- | --- | --- | --- | --- | --- |
| **pDC** | + | CD172a-IgG1  CD205-IgG2b  CD11c-IgM | IgG1-PE-Cy7  IgG2b-AF647  IgM-PerCP-Cy5 | + | CD13-IgG1-AF488  CD86-IgG1-PE | + | TNF-IgG2a | IgG2a-APC-Cy7 |
| **Th2/Tc2** | + | CD8-IgG1  CD4-IgG2a  CD25-IgG3 | IgG1-PE-Cy7  IgG2a-APC-Cy7  IgG3- PerCP-Cy5.5 | + | CD45RO-IgG3-PE | + | IL-4-IgG2a-FITC  IFN-γ-IgG1-AF647 | - |
| **γδ T** | + | CD8-IgG1  CD4-IgG2a  CD25-IgG3 | IgG1-PE-Cy7  IgG2a-APCy-Cy7  IgG3- PerCP-Cy5.5 | + | WG1-IgG1-FITC | + | IL17A-IgG1-PE  IFN-γ-IgG1-AF647 | - |
| **Tregs** | + | CD4-IgG2a  CD25-IgG3 | IgG2a-APC-Cy7  IgG3- PerCP-Cy5.5 | + | CD45RO-IgG3-PE | + | TGF-β-IgG1  IL10-IgG2b | IgG1-PE-Cy7  IgG2b-AF647  FOXP3-IgG2a-AF488 |
