# Supplementary material for: The specific features of the developing T cell compartment of the neonatal lung are a determinant of respiratory syncytial virus immunopathogenesis
Source: PLoS Pathog. 2021 Apr 28;17(4):e1009529. doi: 10.1371/journal.ppat.1009529 (PMC8109812; doi:10.1371/journal.ppat.1009529)
Supplement: Attachment — Submitted filename: Demoulins et al_Reviewers Comments_Revisions.docx [file ppat.1009529.s012.docx]

We thank the Editorial Board of *PLOS Pathogens* for giving us the opportunity to submit a revised version of our manuscript. With the help of the Reviewer’s comments/suggestions and additional experiments/data, we think that the quality of our manuscript has significantly improved. Please find below our point-to-point answers to each comment in blue print. All the changes made in the original manuscript are also highlighted in blue print.

**PART I – SUMMARY**

**Reviewer #1**

The manuscript from Demoulins, et al. utilizes a lamb model of neonatal RSV infection to examine the T cell response to the virus. The advantage of the lamb model is that the lungs of lambs are similar to human lungs and so are a better model than other animal models that are commonly used for understanding RSV infection. The authors infected lambs a week after birth and followed them for 6 weeks. This allowed for data to be obtained at various points during development, a strength of the study. Weaknesses in data presentation including only showing a representative FACS plot and not tabulated data and using Pie charts without showing means and errors. Statistical analysis is also not appropriate for all of the data.

We thank the Reviewer for the helpful suggestions. Accordingly, we have improved the representation of our data and worked on the statistics (please see “Part II and Part III for extensive replies).

**Reviewer #2**

The overall objective of this manuscript is to characterize a neonatal lamb model of respiratory syncytial virus (RSV) infection. The rationale for conducting the study is based upon distinct clinical outcomes of RSV infection in young children relative to adults. Although both children and adults can become infected with RSV, severity of symptoms including distal airways inflammation is pronounced during early life. Increased susceptibility to severe RSV disease in young children is thought to be mediated by an immature immune system, but little is known about the developing immunity in the respiratory tract.

To address this gap in knowledge, the authors of the study infected 6 day old neonatal lambs with a human RSV strain (n=6 at each timepoint). The outcome of infection was compared with mock (PBS) infected neonatal lambs (n=3 at each timepoint). Cellular and humeral immune responses were compared with uninfected adults (n=3 at each timepoint). For a portion of the study, the neonatal cellular immune response to RSV infection was compared in a small cohort of infected adults (n=3 at 6 and 14 d.p.i.). Two different strains of RSV virus were used, including the ATCC RSV-A2 strain as well as the RSV-ON1-H1 strain isolated by the authors from an infected child. Strengths of the study include the importance of understanding early life lung immunity, particularly in the context of respiratory viruses. There are several important limitations of the study findings. The primary read out for virus is PCR, however this method does not establish replication. Because TLR3 activation does not require infectious virus, a treatment group with UV inactivated virus would have been an additional important control. The authors do find a number of modest differences in lavage/tissue cell populations, which could be strengthened by statistics.

We are grateful that the Reviewer appreciates the overall quality of our study. We have addressed the concerns of the Reviewer by preforming additional experiments to demonstrate the replication of human RSV in sheep (please see Part II and Part III for further details).

**Reviewer #3**

The study examines an important area i.e. immunity and disease after RSV infection in neonates. The study and model are being sold by the authors as the gold standard and one that properly recapitulates human disease. If this is going to be the case, then the authors need to discuss how the model recapitulates (or does not) the diversity in the response in human infants and children. Not all children have severe disease. Where does the model fail to recapitulate the response of human children undergoing their 1st RSV infection, age 1… 6 months old, etc. Issues of passive Ab and others are not examined. What are the strengths and weaknesses of the model. Percentages of cells rather than cell numbers are examined throughout. Percentages do not always reflect the response as they are dependent on other cell populations remaining similar and total cell count being equal. The data herein suggest those other cell populations are not remaining the same. Thus, interpretation of the percentages is difficult. For many datasets, only a single representative sample is provided. It is unclear how representative these single samples are.

We thank the Reviewer for the helpful recommendations that significantly improve the quality of our manuscript (please see Part II and Part III for further details).

**PART II – MAJOR ISSUES: KEY EXPERIMENTS REQUIRED FOR ACCEPTANCE**

**Reviewer #1**

1. Statistical analysis is described in methods as non-parametric tests between two groups. However, some of the data looks like analysis of variance or the non-parametric equivalent would be more appropriate (Figs 5c and E for example).

As the development of pulmonary immunity is highly dynamic, marked by a strong cellular colonization, we consider that in the first part of the manuscript (Figures 1-5), statistical comparison between groups was appropriated only in the following cases: 1) Adults versus Mock neonates at the initial time point (baseline); 2) RSV A2-infected neonates versus mock neonates at each time points (impact of RSV A2 infection on the neonatal developing T-cell compartment). This is why we employed the Mann-Whitney U test in most analysis. To improve the clarity about which groups are compared, we have reworked all the related Figures (namely **1B**, **1E**, **1F**, **2A**, **2D**, **2E**, **3A**, **3E**, **3G**, **4A**, **4B**, **4D**, **4E**, **4G**, **4H**, **5A**, **5D** and **5E)**. Also, we have specified at the end of each Figure Legend which statistical tool was used.

Concerning the second part of the manuscript (Figures 6-8), the Reviewer is correct that a one-way ANOVA test is more appropriated, given that we are interested in the comparison between all groups. The corrected Figures appear now (namely **6B**, **6D**, **7A**, **7C**, **7D**, **7F**, **7H**, **7J**, **7L**, **7N**, **7P**, **7R**, **8B** and **8D**), as well as the Figure Legend have been clarified. The main text related to this part (**Pages 11-13, lines 260-312**), remains almost unchanged, as statistical re-analysis does not alter the scientific content and conclusions of the article in any way.

2. In the discussion the authors suggest (line 294-6) that pDC might have defects in signaling by RSV or deficiency in cytokine production. Do you have any data on type I interferon production by pDCs?

No validated antibodies against ovine type I IFNs are commercially available, thus preventing this direct investigation. This is now mentioned in the text, as follow: **Page 8, lines 165-6, Results section**: "It should be noted that no Ab for ovine type I IFN detection are available, explaining why we employed instead mRNA expression levels."

3. It is curious that the authors did not discuss the possibility the ILCs could have a role in the TH2 bias they see early after infection. This should at least be part of the discussion if they did not examine them directly.

We thank the Reviewer for this remark. Accordingly, a paragraph dealing with this aspect has been added in the discussion section, as follow: **Page 16, lines 383-9**: " Also, one must consider the key role of type 2 innate lymphoid cells (ILC2s), a scarce innate subset with lymphoid morphology but distinct from T cells, in promoting IL-4 secretion in the BALs of RSV-infected neonates (54, 56, 57). This direct investigation was prevented by the lack of ovine antibodies to constitute the accurate FCM panel for a proper investigation. However, a proportion of IL-4-producing CD4^-^CD8^-^ cells was detected, supporting the notion that ILC2s may participate to the early and robust induction of type 2 cytokines."

**Reviewer #2**

1. It would be helpful to provide more details for the overall experimental design to understand the different animal groups. The schematic in Figure 1 is acceptable but additional information on procedures conducted at each time point presented in an organized fashion (e.g. table that accompanies the figure) would improve the manuscript.

In accordance with this recommendation, we have improved **Figure 1A**. Now is depicted a more exhaustive and comprehensive illustration, comprising additional details about which procedures, as well as the exact time points, were conducted for each animal group. Moreover, a text section in the Figure 1A legend has been added to improve the clarity, as follow: **Page 34, lines 839-43**: "RSV A2-infected animals were injected with 10^8^ PFU of RSV A2. As controls, PBS-injected newborns and non-infected adults were used. Before and after RSV A2 infection, clinical evaluation was assessed daily at early time points (between day 0 and day 6) and twice a week at later time points (between day 7 and day 42). Necropsies were done at days 3, 6, and 14 or 42 p.i."

2. Figure legends could use more detail. It was often difficult to understand how the data were collected and analyzed (both main and supplement figures)

We have elaborated the descriptions, clarified the methodologies and provided more details in all main and supplementary Figure legends.

3. As discussed in the summary, it would be important for the authors to discuss the limitations of the study with regards to viral replication. This text can be included in the discussion.

To demonstrate RSV replication in sheep, we used two additional and independent approaches. First, we used primary ovine well-differentiated airway epithelial cells (WD-AECs) at the air-liquid interface (ALI) challenged with RSV A2. The replication curves are presented in the **new Figure S1A**. We added several portions of text accordingly: **Page 5, lines 99-100:** "Notably, RSV A2 proved to replicate in ovine well-differentiated airway epithelial cell (WD-AEC) cultures, with a peak of infectious release at 72-96 h, as shown in Figure S1A". Notably, these experiments ran on WD-AEC cultures was contributed by **Damian Jandrasits**, who has been added in the author list. Second, we measured the nasal RSV loads using nasal swabs collected during the animal trial. These new data are presented in **new Figure S1B** and described in the results section as follow: **Pages 5-6, lines 107-12:** "RSV A2 loads were followed daily using nasal swabs on a 6 day-duration period (Figure S1B). Shortly after inoculation (day 1-2 p.i., depending on the animal), a peak followed by a rapid decline of virus load was observed; this first peak was due to a caught reflex triggered by the intra-tracheal injection. Remarkably, RSV loads subsequently re-increased to achieve a second peak in 5 out of 6 animals (day 2-5 p.i., depending on the animal), indicating the capacity of RSV A2 to replicate in our experimental model." A method section was added to describe the establishment of ovine WD-AECs (**Pages 18-19, lines 439-66**) and the methodology of nasal swabs collection and RSV loads quantification (**Page 20, lines 490-1;** **Page 22, lines 543**).

**Reviewer #3**

1. In Figure 1, why is there a positive viral titer in the mock group? Is there transfer of the virus in cohoused animals that complicates this analysis?

Mock-treated controls (adults and neonates) and RSV-infected animals were housed in separated stables. Furthermore, the clinical evaluations or necropsies were always done in the "Mock stable" first, and then in the "RSV-infected stable". However, we agree that we should have considered more carefully this RSV-positive mock sample (Sample 1, mRNA extraction 2019). Since we had two distinct lung samples per animal for each time point, we performed a total RNA extraction and the RSV quantification by RT-qPCR again on this sample (Sample 1, mRNA extraction 2021) and for the remaining lung sample (Sample 2, mRNA extraction 2021). We found RSV A2 undetectable in the two new total RNA extractions as shown in the figure below.

Sample 1

Sample 1

Sample 2

This indicates that the positive RSV signal presented in Figure 1B was probably due to a cross-contamination occurring during the RNA extraction procedure and not an undesired contamination in the stables. Thus, we propose to revise the **Figure 1B, "Lung panel"** with the new measurements.

What are the samples from in Fig 1C? Is this only the neonates? The RSV+ type I pneumocytes and macrophages should be indicated in these images. How were the macrophages and type I pneumocytes identified? In addition to these provided images the results of several samples should be quantified.

Based on these pictures, we agree that alveolar macrophages and type 2 pneumocytes cannot clearly be distinguished morphologically. We therefore adjusted the text and figure legends. In this work, histology was used as a qualitative method to confirm or rule out presence of virus-induced lesions in the lung. Representative samples from macroscopically changed areas and control animals were chosen. Thus, we believe that histological quantification of lesions is not appropriate.

Figure 1E, so were all the healthy adults screened to be RSV negative in order to be included in that study group. It is strange that the mother in question that passively transferred Ab to the neonate appears to have been positive. What was the seropositivity of the adult animals in the facility? This also introduces an additional important parameter to the study/model for it to be translational. How does the current model reflect (or not) human infants which may have passive antibody from their mothers. It appears the response in the 2 animals that had Ab may result in lower titers at day 13/14.

In the previous version of the manuscript, only animal groups euthanized at days 14 and 42 p.i. were screened for the initial presence of RSV NAbs at baseline. Indeed, we aimed at using serum-neutralization assay to show the induction of a humoral response, reason why we omitted groups euthanized at day 3 and 6 p.i (too early time points to detect an antibody response). However, we agree of the importance to screen those animals as well. We performed a new experiment, which is represented in **new Figure S3**. Also, we adjusted the text in the results section accordingly: **Page 6, lines 125-9:** "Next, we monitored the presence of RSV neutralizing antibodies (NAbs) in the serum of all animals before initiating the trial (animal groups euthanized at days 3 and 6: Figure S3; animal groups euthanized at days 14 and 42: Figure S4). Besides a sibling (see below), none of them showed neutralizing activity. Then, in the serum of healthy adults and mock neonates, no RSV NAbs induction were detected up to 13-14 days p.i. (Figures 1E, S4)." Regarding the siblings presenting a RSV A2 neutralization prior infection, we have included potential hypothesis: **Page 6, lines 133-5**: "Whether these NAbs were induced following infection by bovine RSV and cross-reacted with human RSV or the result of a random V-D-J recombination that specifically recognizes human RSV epitopes, was out of the scope of the present study." Concerning the passive antibody transmission by the mother, we think that although this analysis could potentially provide some interesting insights, we feel this is out of the scope of the present study.

Figure 1G, the figure this reviewer had to examine does not contain any data for days 3, 6, and 42 only data at day 14. This needs to be fixed.

What the Reviewer means is unclear to us, since the Figure 1G displays the results obtained for all individual neonates included in the trial (i.e. BAL fluid fraction collected at days 3, 6, 14 and 42 p.i.). This confusion was probably due to the lack of information in the figure legend, which has now been corrected.

2. Why do mock levels of gd T cells also drop in the BAL in Figure 2A. In addition to the representative samples shown, data in Figure 2C-E for all animals examined should be shown in graphs as in Fig 2A and the results analyzed by statistical analysis.

The drop observed in Figure 2A, as well as in the **new Figure S6C,** does not reach statistical significance and as such, was not considered.

Why does the IL-17 level in the mock increase at day 42 in Figure 2E?

A slight increase is indeed observed in Mock neonates euthanized at day 42, to reach the very low levels observed in healthy adults. We think that this is a spontaneous low production due to the cell isolation procedure, and we think that this is negligible compared to the high levels of IL-17 detected in RSV A2-infected adults (Figure 7E)

If IFNg is being analyzed the percentages should be provided.

As recommended by the Reviewer, the percentages have been added in the related Figures of this revised version, namely **Figures 3F**, **4C**, **4F**, **7E**, **7I** and **7M**.

3. Data in Fig 3 A/B should be shown as cell numbers rather the percentages, as the percentages can vary based on changes in other cell populations. 100 T cells among 1000 total cells and 100 T cells among 10,000 cells is still only 100 T cells in the tissue. In Figs 2 and S4/5 the authors argue for other cell populations to be changed in the RSV infected neonates. Thus, the percentages here are very difficult to interpret as these other populations are changing. The text comments on IFNg not being different but no numbers for IFNg+ events are provided on the plots to allow the reader to assess this. Likewise numbers should be provided in Fig 3D/F due to the fact the number of T cell in each group may not be equivalent.

We would like to point that we are fully aware of potential issues concerning percentage versus absolute number. However, for our immune subset counting, we are dealing with the [percentage of total events acquired], and not the [percentage of the parent population]. This approach minors the influence of the variation in frequency of other cell subsets. We decided to use this readout to quantify the cellularities for two reasons. First, BAL methodology in large mammal is tricky, requiring to take out the trachea, and collect the cells with large PBS volumes. Although the same procedure was strictly applied to all animals, we cannot guaranty that the whole cells were collected with the same efficiency (as previously mentioned by the reviewer, we are dealing with small numbers of cell subsets). Second, cell counting was done under the microscope with Trypan blue exclusion, and human factors can add to the BAL procedure-related variability. By proceeding with the [absolute counts] instead of [percentage of total events], we are convinced to introduce bias and to increase disparity that simply do not reflect the reality. In contrast, we consider the [percentage of total events] as an accurate and unbiased picture of what is happening in the bronchoalveolar space, which is why we decided to show this readout rather than the absolute counts.

However, since this issue is of particular importance, we are now providing a **new Figure S6**, which displays the BAL cellularity, as well as the absolute counts for all animals of all cell subsets included in the study (pDCs, γδ T cells, CD4^+^ and CD8^+^ T cells and Tregs). And despite the disparity factor added due to the above-mentioned reasons (BAL procedure, manual cell counting), the same differences were obtained, with the exception of the γδ T-cell subset, for which significance is lost although the trends is still observable. In **Figure S6G**, we show that the decrease of γδ T cells in Figure 3A is not due to the variation in frequency of others immune cell subsets. This is also clarified in the result section as follow: **Page 8, lines 181-4**: "Importantly, the decrease of γδ T cells was not the indirect consequence of the relative increase of another cell subset, since no negative correlation was found when the % of total events of γδ T cells was plotted with % of total events of pDCs, CD4^+^ T cells, CD8^+^ T cells and Tregs (Figure S6G)." In addition, we are now providing the absolute counts of IL4^+^ CD4^+^ T cells (**new Figure 4E**), IL4^+^ CD8^+^ T cells (**new Figure 4H**), as well as a comparison those two entities (**new Figure 4I**). The related sections in the text has been reworked accordingly: **Page 9, lines 213-6**: "Upon infection, this analysis revealed a trend towards an increase in the frequency of those Th2 cells, whereas there was no consistent influence on IFN-γ induction (Figures 4C (representative contour plots), 4D (interleaved scatters integrating all animals) and 4E (absolute counts))." **Page 10, lines 223-6**:"…(Figure 4F (representative contour plots), 4G (interleaved scatters integrating all animals) and 4H (absolute counts)). When we compared the absolute numbers of recruited Th2 and Tc2 cells in BALs, we found that the IL-4 response at days 6-14 p.i. was mainly driven by the Tc2 cells (Figure 4I)." Finally, we are now providing the absolute counts of TGF-β^+^ Tregs (**new Figure 5E**), which proves to be significant as well.

Finally, it has become clear in many experimental models that many of the T cells particularly in the uninfected individuals are in the circulation and not in the lung airways or interstitium. Thus many groups have begun to label cells in the circulation with Ab in order to accurately measure only those cells in the lung interstitium and airways. The important T cells found within the interstitium, and contribute to disease control and immunopathology, are not examined herein.

This was also one of our thoughts, when we ran a pilot experiment on 12 neonatal lambs. However, our initial observations in the peripheral blood were moderated at best, prompting us to emphasize the respiratory compartment and draining LNs for the two animal trials reported in the present study.

**PART III – MINOR ISSUES: EDITORIAL AND DATA PRESENTATION MODIFICATIONS**

**Reviewer #1**

1. Data are interesting, but some of it needs to be in a different format to make it clear whether there are actually differences between groups. The authors chose to put what they call significant data on pDCs in supplemental figure 5 rather than show the data in the body of the paper. This is not helpful and the data should be in the main body of the paper.

As recommended by the Reviewer, we have moved the pDC results to the manuscript body and to **Figure 2**.

2. Figure 2 shows FACS plots without showing all of the data in graph form. This could be in supplemental data since the authors state it is not statistically significant. Contour plots in Fig 5 F,I, and L show what are probably percentages in lower right quadrants with an asterisk associated with them. There is no indication from the figure legends what these numbers are. Are they means? If means, what are the standard errors and how many individuals were used for the calculation? This is an example of using graphs to show these data. Several figures have Pie charts in them which are unhelpful from the standpoint that the reader can’t see standard error and not statistical analysis is indicated. I you want to keep the pie charts for the visual they provide then the means and standard errors should be in the supplemental data or in the text.

We understand the concerns of the Reviewer and a panel of new sub-figures has been added, either in the main figures (**new Figures 3E**, **3G**, **4K**, **5D**, **7C**, **7F**, **7J**, **7N** and **7R**) or in the supplementary figures (**new Figure S9**). Moreover, we have clarified the methodologies and provided more details in all main and supplementary Figure legends.

3. Symbols used in figure 5 (purple) appeared there suddenly without any explanation. Fig 6B does not indicate which viral strain goes with which set of data.

We added an **illustration as Figure 6A,** explaining in detail the experimental design, procedures, and time points for each animal group of the data reported in Figures 6-8. Also, a paragraph has been added in the Figure 6 legend, as follow: **Pages 37, lines 943-6**: "Experimental design of trans-tracheal RSV A2 or RSV-ON1-H1 infections. Animals were injected with 10^8^ PFU (RSV A2) or 10^7^ PFU (RSV-ON1-H1). As controls, PBS-injected newborns or non-infected adults were used. Before and after RSV infection, clinical evaluation was assessed by a veterinarian, every 3 days. Necropsies were done at days 6 or 14." Finally, Figure 6B (now **8B**) has been improved to show the missing information, as well as the exact sequence of the primers used is now provided in the Material and Methods section: **Page 22, lines 557-9**: "For the comparison between RSV A2 and RSV-ON1-H1, the following panRSV primers were used…."

**Reviewer #2**

1. What is the basis for the dose of virus? It is also unclear which animals received the A2 strain versus H1 strain. Was the H1 strain exclusively used for Figure 6? What was the rationale for selecting the two strains?

The basis of the dose of virus comes from published studies done by the group who established the lamb model of human RSV infection, namely the group of Prof. Mark R. Ackermann (Iowa State University, cited in our References #28-30). These investigators typically use RSV doses comprised between 10^7^ and 5x10^8^ PFU per animal. To establish the lamb model in our facility, we applied this range of viral doses in a set of pilot infections on a small cohort of lambs (3 mock and 9 RSV-infected lambs).

To clearly identify which animal received A2 or H1 strain, we have added a **new Figure 6A** explaining in detail the experimental design for each group of the data reported in Figures 6-8. Also, a paragraph has been added in the Figure legend, as follow: **Page 37, lines 943-6**: " Experimental design of the trans-tracheal RSV A2 or RSV-ON1-H1 infection. Animals were injected with 10^8^ PFU (RSV A2) or 10^7^ PFU (RSV-ON1-H1). As controls, PBS-injected newborns or non-infected adults were used. Before and after RSV infection, clinical evaluation was assessed by a veterinarian, every 3 days. Necropsies were done at days 6 or 14." Also, the text and figures have been examined closely, so that now it is always clear whether A2 or H1 strain was used. Finally, we agree that we should have explained the rational of selecting the H1 strain. This is now specified in the text of the results section as follow: **Page 12-13, lines 298-302:** "While immune-mediated mechanisms are central determinants of RSV severity, there are indications that viral factors play also a role (35). We evaluated the pulmonary T-cell response in neonates infected with RSV A2, a well characterized prototypical strain of RSV, in comparison to the RSV-ON1-H1, a primary clinical isolate which was the major genotype circulating in central Europe during the RSV seasons of 2012 to 2017 (Figure 6A) (36)."

2. Is there evidence of viral replication? This is critical to confirm the immune outcomes and pathology are due to infection versus an inflammatory response to the inactivated virus. PCR measures are informative but are not sufficient to confirm infection; plaque assays are needed. As an alternative, comparative analysis using mock treatment with UV inactivated virus would confirm infection as opposed to TLR mediated inflammation.

We addressed this point by preforming two additional experiments and we kindly refer the Reviewer to our reply of a related comment (Part II Major Issue, Reviewer #2, reply #3).

3. It is surprising that no differences in blood PMN were detected giving the apparent detection of inflammation in the lung. Are PMN readily visible in the alveolar compartment of RSV infected animals?

The role of PMNs is well established in RSV disease and was thus not the focus of your study. As a consequence, we didn’t design an FCM strategy to detect PMNs. However, in the lamb model of RSV infection, infiltration of PMNs 3 to 6 days post infection is described by the group who established the model when using RSV A2 or a clinical isolate as challenge viruses (PMIDs 19659901, 0935230, 24804166). In line with this, we observed histologically infiltration of inflammatory cells mainly 3-6 days post-infection. This qualitative evaluation is described in the results section as follow: **Page 6, lines 120-3**: “Histological lesions, present at days 3-6 p.i consisted of multifocal-coalescing areas of lymphocytic and histiocytic infiltrates within interalveolar septae (interstitial pneumonia) and accumulation of inflammatory cells within alveolar spaces and bronchioles (bronchiolitis) (Figure 1D).”

4. Were cell counts and basic cell phenotyping conducted on BAL samples (e.g. cytospin of lavage cells)? This would provide quantitative measures of key cell types responding to the RSV, including macrophages, neutrophils, eosinophils, and lymphocytes.

Total cell counts were done during the euthanasia and are now shown in **new Figures S6A** and **S8**. Also, based on the recommendation of Reviewer #3, we are now providing the absolute numbers of the different subsets of the study (**Figures S6B-F**, **4E** and **4H**). Unfortunately, we didn’t perform cytospin evaluation of BAL cells and restricted our analysis to the histological evaluation. We kindly refer the Reviewer to our reply above (point 3).

5. The authors show an increase in the BAL pDC population with RSV but the numbers are very low. While it would not be surprising to find low pDC frequency in BAL, the overwhelming presence of macrophages in BAL samples may create autofluorescence background issues by FACS. Autofluorescence can be further exacerbated when using reagents that are not directly conjugated with fluorochromes. What controls were used to validate the FACS panel for pDC?

Notably, no antibodies against ovine type I IFNs are commercially available, which would have made the definitive tool to validate ovine pDCs. This limitation is now mentioned in the text of the results section, as follow: **Page 8, lines 165-6**: "It should be noted that no Ab for ovine type I IFN detection are available, explaining why we employed instead mRNA expression levels." Notably, our combination staining panel for the identification of ovine pDCs was established in collaboration with the group of Prof. Artur Summerfield (University of Bern, Switzerland), who is internationally acknowledged for his expertise in the field of DC biology in large animals. The phenotyping of DC subsets in ruminants, including pDCs, was published recently by Prof. Summerfield (PMID 30425716). Finally, the most striking observation related to pDC activation was the CD86 upregulation, which is a commercial directly conjugated antibody, as shown in Table S2.

6. It is difficult to interpret Figure 2B. Are the authors inferring that gamma delta T cells are highest when there is more overall cell death due to RSV? Why would gamma delta T cells decline?

Although this analysis could potentially provide some interesting insights, we feel that this is out of the scope of our study. Nevertheless, the **new Figure 3E** helps to better understand, as it clearly shows that the γδ T cell disappearance occurs mainly within the WC1^+^CD25^-^ subset, suggesting a retention at this immature stage.

7. Lines 176-177, the authors state “an unexpected reduction of CD4+ T-cell recruitment was seen as soon as 14 days p.i.”. It this reduction significantly different? The change appears modest at best.

We agree with the Reviewer. We have toned down the statement accordingly: **Page 9, lines 203-5**: "An unexpected reduction of CD4^+^ T-cell recruitment was seen as soon as 14 days p.i., although not significant (% of total event at day 14 p.i. versus day 6 p.i.: p=0.0649)."

8. For the study described in Figure 5, did the adults and neonates receive the same dose/volume of virus? If so, what is the difference in lung volume between the two ages and could the lack of inflammation in adults be explained by dilution effect?

The reviewer is raising a valuable point. While we didn’t measure precisely the lung volumes, to obtain BALs we consistently used 200-300ml of PBS for neonates and 800-1000ml for adult animals. Nevertheless, we decided to inject the same dose and volume of virus in adults and neonates. This decision was based on the results obtained in the first animal trial (Figure 1A) and a pilot experiment done on 12 neonatal lambs. By considering those two experiments, we could determine that 10^7^ or 10^8^ PFU made no differences in terms of pulmonary viral loads and lung lesions in neonates. Therefore, we think that a 2-4 fold increase of RSV A2 dose in adults to take in account the higher lung volume, would have made no difference, except introducing a bias difficult to evaluate.

**Reviewer #3**

1. The uniqueness of this work is being oversold. There have been other studies on T cell immunity and neonatal responses. Thus, the authors selling of the work as “unprecedented” (line 92) and other similar descriptions in the manuscript seems as though the authors are unaware of prior studies some of which they cite.

We agree that the wording "state of the art" model is unnecessarily polemic, which is why we have replaced by "translational" model, which sounds less partial. Also, in accordance we the recommendation, we rewrote this sentence in the introduction section, which is now as follow: **Page 5, lines 91-4**: "Moreover, by peribronchial lymph nodes (LNs), lung, and bronchoalveolar space sampling following RSV infection, we assessed the potential contributors of the host neonatal T cell compartment (i.e., CD4 and CD8 T cells, γδ T cells and Tregs) over the course of RSV disease". Otherwise, from a review of the literature, we think that our results are objectively discussed, and that we provide novel insight in the field of early life lung immunity, particularly in the context of respiratory virus infection.

2. The figure design in many places is not clear, well labeled or adequately explained in the legends/text.

In accordance with this comment, we have clarified the methodologies and provided more details in all main and supplementary Figures.

3. How are the responses in the 2 animals with passive antibody, or other animals with passive Ab, altered in the subsequent figures and analysis?

This relates partially to similar comments from same Reviewer, Major Issue #3. In the previous version of the manuscript, only animal groups euthanized at days 14 and 42 p.i. were screened for the initial presence of RSV NAbs at the baseline. In this revised version, animal groups euthanized at days 3 and 6 p.i. were screened as well, and we found no other neonates with passive Ab (**new Figure S3)**. Now regarding the siblings presenting an RSV A2 neutralization prior infection, we do not think that this alter the figure and analysis, given that all RSV-infected neonates euthanized at day 42 p.i. exhibit very few remaining signs of immune activation, as well as a tight grouping of almost all experimental readouts is seen.

4. Data in S4 and S5 should be included in the paper and not be supplemental.

Figure S4 has been moved and is now **Figure 2**. For figure S5, we think that we have already a consequent amount of data presented in the main figures and we would like that the gating strategies remains in the supplementary material.

5. Fig 4 A, please see comments about the need to distinguish cells in the lungs vs circulation above.

This relates to similar comments from same Reviewer, Major Issue #3, last point.

6. What is the basis for the statement on line 235-6 that the response is tightly regulated?

We agree that this sentence was an overstatement, given that the percentage of CD86^+^ pDCs in RSV A2-infected adults at day 14 p.i. remained superior to what calculated in healthy adults (Figure 2C). Therefore, we have decided to remove this sentence.

7. Overall day 0 or controls should be shown for all groups in Fig 5. Again all data should be shown for figures such as Fig 5D/F/G/I/J/L/M not just representative samples. Statistical analysis should be undertaken.

In accordance with the principles of the 3Rs, we have limited the number of Mock neonates used as we collected enough data with prior experiments. The goal of the animal trial presented now in Figures 6-8 was to compare the cellular immune response upon RSV infection in adults vs. neonates (Figure 6, 7) or in neonates infected with RSV A2 vs. RSV-ON1-H1 (Figure 8). In this context, the "grey boxes" appeared to us to be the best way to summarize the results collected for Mock neonates and Healthy adults in our various animal trials. Concerning the Figures showing representative samples, we understand the concern of the Reviewer and a panel of new sub-figures has thus been added, with statistics, either in the main figures (**new Figures 7C**, **7F**, **7J**, **7N** and **7R**) or in the supplementary figures (**new Figure S9**). We also applied the same approach and show now all the data in the **new Figure 3E, 3G, 4K, 5D and 5E**.

8. Cell numbers not just percentages should be shown in Fig 6 (see issues with only examining the % that are discussed above).

This relates to similar comments from same Reviewer, Major Issue #3.

Finally, in parallel to the experiments ran to answer the questions raised by the 3 Reviewers, we have produced new data which we would like to incorporate in the present manuscript: **new Figure 6D**: Adults and neonates produced RSV A2 NAbs following infection.
