# Supplementary material for: The specific features of the developing T cell compartment of the neonatal lung are a determinant of respiratory syncytial virus immunopathogenesis
Source: PLoS Pathog. 2021 Apr 28;17(4):e1009529. doi: 10.1371/journal.ppat.1009529 (PMC8109812; doi:10.1371/journal.ppat.1009529)
Supplement: Attachment — Submitted filename: Demoulins et al_Reviewers Comments_Revision2.docx [file ppat.1009529.s013.docx]

We thank the Editorial Board of *PLOS Pathogens* for giving us the opportunity to submit a second revised version of our manuscript. With the help of the Reviewer’s comments, we think that the quality of our manuscript has improved and we hope that our study is now ready for acceptance in *PLOS Pathogens*. Please find below our point-to-point answers to each comment in blue print. All the changes made in the manuscript are also highlighted in blue print.

**Part I - Summary**
Please use this section to discuss strengths/weaknesses of study, novelty/significance, general execution and scholarship.

Reviewer #1: The authors have extensively addressed the concerns of the reviewers including adding more data and cleaning up the presentation and statistical analysis of data.

We are glad that Reviewer finds the changes of the revised version satisfactory.

Reviewer #2: The overall objective of this revised manuscript is to investigate the pulmonary T cell response to respiratory syncytial virus (RSV) infection using a neonatal lamb model. RSV is a highly relevant infection in young children that can result in hospitalization and prevention is limited to prophylaxis. The authors conduct immune cell profiling of normal lungs and compare with lungs following viral infection, with the rationale that the T cell response may elicit a distinct signature with RSV. Neonatal responses are compared with adult animal responses. In addition, two different variants of RSV are tested to assess for differences in ability to promote local immune responses. Strengths of the manuscript include the overall clinical relevance of understanding the distinct pulmonary immune response to RSV within pediatric populations and the use of a large animal model. As written, there are a number of points within the manuscript that appear to overstate the outcomes of each experiment.

We are grateful that Reviewer appreciates the overall improvement of our revised manuscript. We have addressed the additional concerns of the Reviewer (please see below, Part III).

**Part II – Major Issues: Key Experiments Required for Acceptance**
Please use this section to detail the key new experiments or modifications of existing experiments that should be absolutely required to validate study conclusions. Generally, there should be no more than 3 such required experiments or major modifications for a "Major Revision" recommendation. If more than 3 experiments are necessary to validate the study conclusions, then you are encouraged to recommend "Reject".

Reviewer #1: None.

Reviewer #2: (No Response)

**Part III – Minor Issues: Editorial and Data Presentation Modifications**
Please use this section for editorial suggestions as well as relatively minor modifications of existing data that would enhance clarity.

**Reviewer #1**

Line 265 should indicate the strain of virus being used is a clinical strain rather than referring the reader to the next section.

As recommended by the Reviewer, it is now indicated, as follow: **Pages 11, lines 264-6**: "Additionally, neonates were infected with RSV-ON1-H1 strain, a primary clinical isolate recently circulating in central Europe (36)”.

**Reviewer #2**

1. Lines 67-69 Upon stimulation neonatal T cells tend to differentiate into regulatory T cells (Tregs), to facilitate self-tolerance to developing organs. This sentence needs to be rephrased. As written, it suggests that most neonatal T cells are Tregs. While it is true that neonates have higher frequencies of Tregs, this is not the majority of the T cell population. In the human fetus, there are indeed a higher frequency of Tregs (about 15%) but infants and adults have about 5% in the CD4+ cell population.

We agree with the Reviewer and have reworded the text accordingly: **Page 4, lines 66-8**: " Upon stimulation, neonates have a higher frequency of T cells that differentiate into regulatory T cells (Tregs) compared to adults, to facilitate self-tolerance to developing organs (10)." **Page 15, lines 366-7**: "As fetal CD4^+^ T cells tend more to differentiate into Tregs after stimulation than in adults (10), …"

2. For Figure 1B, there is no description of what the data on the left represent. Mock? Adults?

This lacking description has been added in the legends of Figures 1-5: Adu., healthy adults.

Moreover, by checking carefully the Figures, we noticed that the "Adu." abbreviation was lacking in **Figures 2D** and **5D**. This has been added and the corrected Figures provided.

3. How are the data in Figure 1G quantified? Are there replicates?

Those missing information's have been added in the legend of Figure 1, as follow: **Page 34, lines 86970.** " Cytokine concentrations were quantified with a multiplex assay and a single measurement was done per animal tested."

4. Lines 192-193 Finally, we demonstrated the inability of neonatal gamma delta cells to produce detectable levels of IL-17 (Figure 3F, G). This statement is inconsistent with the figure. IL-17 is detectable at day 42 in the neonates.

The following sentence, initially localized at the end of the manuscript in the Material and Methods section, was moved at the very beginning of the Results section: **Pages 5, lines 104-6**: "The developmental age of sheep does not follow a linear curve compared to humans but in terms of lung development, animals euthanized at day 3, 6, and 14 post-infection (p.i.) can be considered as infants and at day 42 as adolescents (31)". With this text rearrangement, it is now clear to the reader that IL-17 detection at day 42 cannot be considered as an observation applicable to neonates. Moreover, a clarification has been added: **Pages 9, lines 192-4**: "Finally, we demonstrated the inability of γδ T cells to produce detectable levels of IL-17 during the neonatal period (days 3-14 p.i.; Figure 3F, G)."

5. The authors make the statement that “neonatal RSV A2 infection induces a fast and massive Tc2 influx in the bronchoalveolar space”. However, there does not appear to be a difference in total cell numbers (Figure S8) It is possible that populations may shift over time, such that macrophage numbers are reduced in response to the virus.

As shown along our manuscript, the cell subsets from adaptive responses are low in frequencies and absolute counts in early life, meaning that even a huge variation at "the Tc2 level" will not – or will poorly – influence the total cellularity of the bronchoalveolar space (at this stage mainly composed of alveolar macrophages). Based on the Figures 4B, 4H and S6E, we think that we are correct with our statement about a "fast and massive Tc2 influx …" following RSV infection. However, we have reworded the title in the Results section as follow: **Page 9, lines 196:** “Neonatal RSV A2 infection induces a fast Tc2 influx in the bronchoalveolar space”.

6. For Figure 7G, what does WC1 represent?

We are sorry for this omission. A reference is now provided for the readership who is not familiar with our model: **Page 35, lines 900-1**: "WC1 is highly expressed in ruminant γδ T cells and is commonly used as a specific marker of this subset (67)."

7. Lines 281-282 From these results, we could ascertain that neonates lack a mature and functional gamma delta T cell. This statement is not reflective of the reported data. Gamma delta cells are detected in the neonate lung lavage but appear to decline following viral infection and therefore produce less IL-17.

This relates partially to similar comments from the same Reviewer, point 4. To avoid any misinterpretation, we are now indicating in brackets the time points concerned by this statement: **Page 12, lines 279**: "[…] suggesting that IL-17-mediated immunity is deficient in early life (days 3-14 p.i.; Figure 7D, E, F) (15)."

8. Lines 283-285 The percentage of IL-4-producing cells was very low…(Figure 7I, J).
This statement is inconsistent with the data shown in Figure 7I and J.

We have modified the sentence, so that it does now reflect what is depicted in Figure 7I and 7J: **Page 12, lines 284-6**: "The percentages of IL-4-producing cells were far from achieving the magnitude observed in neonates, and RSV infection had no consistent influence on IFN-γ induction (Figure 7I, J)."

9. Lines 290-292 From these results, it became clear that the inaptitude of neonates to mount an effector T cell response is tentatively substituted by a fast and massive Th2/Tc2 cell recruitment.
This statement is not supported by the data. What is the evidence that the effector T cell response is ineffective, particularly in light of comparable generation of neutralizing antibodies between neonates and adults (Figure 6D)

We agree that this conclusion was too speculative. In accordance with this remark, we have reworked this section as follow: **Page 12, lines 292-5**: "From these results, it is likely that the inaptitude of neonates to mount a classical effector T cell response is tentatively substituted by a Th2/Tc2 cell recruitment. To elucidate this point, the follow-up of the present work will consist in evaluating the RSV-specificity of this neonatal type 2 responses."

10. Line 292 …we failed to detect any T reg recruitment
This statement is not supported by the data (Figure 5A)

We agree that the sentence lacked of clarity. We have thus elaborated the related part, as follow: **Page 12, lines 295-8**: "Finally, we evaluated the Treg compartment in adults. Unlike our previous observations in neonates, we failed to detect any Treg recruitment in the bronchoalveolar space of infected adults, at least up to day 14 p.i."

11. For Figure 8, authors report that the T cell response is a correlate of severity of lung inflammation in association with RSV strains. There appears to be trend toward increased viral replication with RSV A2, but there are no statistical differences A2 and ON1-H1. It’s difficult to make a conclusive statement about the magnitude of the T cell responses from these data because of the small sample size, unless T cell numbers can be quantitatively correlated with inflammation (e.g. tissue neutrophil numbers).

We have expanded the descriptions, clarified some results and attenuated some conclusions, as follow: **Page 13, lines 308-9**: "Whereas infection of neonates with RSV-ON1-H1 strain led to a milder disease, as indicated by milder histological lesions (Figure 8A), […]"

**Page 13, lines 311-2**: "Effectively, all T cell-related responses were either attenuated or displayed a trend towards attenuation."

**Page 13, lines 312-4**: "We did not observe a γδ T cell depletion, and the CD8^+^ T cell recruitment was delayed (requiring 14 days p.i. to exceed the limits of the Mock grey zone, instead of 6 days p.i. in RSV A2-infected neonates)."

**Page 13, lines 314-5**: "Furthermore, the Treg recruitment was significantly lower compared to RSV A2 infection (Figure 8C)."

By considering the results in Figure 8 and the text modifications, we think that the statement about the magnitude of the T cell responses is appropriate.
